# Scientometric evaluation of 100-year history of *Poultry Science* (1921–2020)

**DOI:** 10.1016/j.psj.2022.102134

**Published:** 2022-08-13

**Authors:** Esmaeil Vaziri, Ali Maghsoudi, Mansoureh Feizabadi, Hadi Faraji-Arough, Mohammad Rokouei

**Affiliations:** ⁎ Department of Information Science and Knowledge Studies, Faculty of Humanities, University of Zabol, Zabol, Iran; † Department of Animal Science, Faculty of Agriculture, University of Zabol, Zabol, Iran; ‡ Department of Animal Science, Faculty of Agriculture, Tarbiat Modares University, Tehran, Iran; § Department of Information Science and Knowledge Studies, School of Medicine, Sabzevar University of Medical Sciences, Sabzevar, Iran; # Department of Ostrich, Special Domestic Animals Institute, Research Institute of Zabol, Zabol, Iran

**Keywords:** content analyses, subject areas, collaboration, research fronts, scientometrics

## Abstract

To have a better contribution to the poultry production community, the Poultry Science Association founded journals including *Poultry Science* (**PS**) at 1921. Now, after 100 yr of publishing, PS ranks between the top 10 journals in the category of “agriculture, dairy, and animal science”. One hundred years after publishing the first paper in PS, the poultry industry has been completely revolutionized. Hence, it will be interesting to establish scientometrics study of the PS development during the last century. Therefore, based on findings of the current study, among countries/authors’ collaborations, future research fronts, and possibility of hot topics in the coming years may be predictable. Accordingly, a total of 22,451 articles were retrieved. For content analyses, according to the PS categorization for subject areas, 14 different subject areas were developed, including “behavior, breeding and quantitative genetics, education and extension, health and welfare, immunology, management and environment, metabolism and nutrition, microbiology and virology, modeling, molecular biology, physiology and anatomy, production, products, processing and marketing, and reproduction”. Considering the 100-yr of PS, the most frequent subject area was “nutrition and metabolism” (14,109 articles), and “modeling” (1,114 articles) attracted less scholarly attention. However, considering the last decade (2011–2020), the most important subject area was “molecular biology” (1,420 of 2,466 articles; 57.58%), followed by “modeling” (544 of 1,144 articles; 48.88%). Moreover, the most frequent poultry species/strains were broilers (retrieved in 6,156 articles), followed by laying hens, turkeys, and quail. Considering collaboration of countries and researchers, it can be said that a total number of 108 countries contributed to PS, with the most prolific country being United States (with 9,421 articles; 43.16%), followed by China, Canada, the Netherlands, and Japan. Among the authors, Harms RH (287 articles), and Siegel PB (208) were the most prolific authors, and Siegel PB and Dunnington EA (71 articles) had more collaborations. To study keyword trends, including 3 time periods broilers was the central co-occurrent keyword, while the importance of chickens and turkeys declined during the time. *Salmonella* spp. was a constant representative of poultry microbiology during 100 yr. While “nutrition and metabolism” was the most important subject area, nutrition-related keywords (major items) were not concentrated and co-occurred with a variety of keywords from different subject areas. While “molecular biology” ranked first over the past decade, the importance of “nutrition and metabolism” should not be ignored. In fact, in recent years, molecular basis of the nutrition has been studied. In big-data era and due to developing the molecular biology technologies, it seems that using mathematical modeling and computational methodologies will increase and probably remains as one of the most attractive research areas for scientists at least in the upcoming future decades.

## INTRODUCTION

The Poultry Science Association (**PSA**) is an American non-profit specialized organization founded in 1908, consisting of approximately 1,800 members of educators, scientists, extension specialists, industry researchers, administrators, producers, and university students. The PSA are committed to advancing the poultry industry (Poultry Science, 2021). In order for members of the poultry industry to have a better contribution, the PSA founded two journals including *Poultry Science* (**PS**; formerly known as Journal of the American Association of Instructors and Investigators of Poultry Husbandry), and the *Journal of Applied Poultry Research* (**JAPR**). The year 2020 was the 100th anniversary of PS publication. This is how their commitment is expressed: “*First self-published in 1921, PS is an internationally renowned monthly journal, known as the authoritative source for a broad range of poultry information and high-caliber research. The journal plays a pivotal role in the dissemination of preeminent poultry-related knowledge across all disciplines*” (Poultry, 2021). Accordingly, based on the journal policies, the main subject areas in PS are breeding, genetics, education, production, management, environment, health, behavior, welfare, immunology, molecular biology, metabolism, nutrition, physiology, reproduction, processing, and products. In category of “agriculture, dairy and animal science”, PS ranks between the top-ten journals (highest ranked journal dedicated to publishing poultry research with H-index = 141) (Scientific Journal, 2020), which have a significant role in improving the knowledge in the global poultry industry.

One hundred years after publishing the first paper in PS (Beach, 1921), the poultry industry has been entirely revolutionized, new technologies have been commercialized, modern high-performing commercial genetic lines have been introduced, research institutes/universities have been developed, the scientific areas have extended, and modern people demands have been emerged. Correspondingly, during last 100 yr, PS has played a critical part in the cooperation among research, industry, and market. Hence, it will be interesting to establish a scientometrics study to monitor how PS was the mirror of poultry industry during the last century.

Scientometrics is considered as a text mining-based research methodology. This type of descriptive research is mainly employed for measurement, analyses, and quantification of scientific publications. Scientometricists provide quantitative indices to describe the scientific output of a certain subject area, authors, institutions, and/or countries. Their findings are usually considered practical tools to evaluate scientific activities, leading to the quantifying scientific productions, scientific policy making, drawing scientific collaboration, drawing future scientific trends and research fronts, and mapping of science. To draw historical perspective and future scientific prediction (research fronts), scientometric evaluation of journals has become more common in recent years (Martínez-López et al., 2018; Singh et al., 2020). However, as far as we know, scientometrics in poultry science is rare (Maghsoudi et al., 2020).

Considering the 100th anniversary of the PS, its 100 yr history has not been evaluated yet; however recently the 100 most cited papers in PS were reviewed (Taylor, 2021). To evaluate the chronological history of PS some questions may be asked: Which subject areas are more interesting for researchers? How are the research trends for each poultry species and/or subject areas during the time? What is the contribution of countries and researchers in published articles? How deep is collaboration among countries and researchers? What have been the subject trends of the published articles of PS over time? Hence, scientometrics could help poultry scientists and scientific policymakers to cover research gaps and try to answer these questions quantitively, which are the aims of this study. Based on findings of the current study, possibility of hot topics in the coming years may also be predictable.

## MATERIALS AND METHODS

### Dataset

All the research and review articles published in PS were retrieved from Web of Science (**WoS**) database. Moreover, publications from 1921 to 1944 which were not covered by WoS, were included manually. Therefore, a total of 22,451 articles were retrieved. Eventually, a dataset containing articles from 1921 to 2020 (100 yr) was created including title, abstract (if available), keywords (if available), authors, country, organization, and year of each publication.

### Content Analyses

#### Subject Areas

The main subject areas in PS including “breeding, genetics, education, production, management, environment, health, behavior, welfare, immunology, molecular biology, metabolism, nutrition, physiology, reproduction, processing, and products”, are listed in PS website (Poultry, 2021). For content analyses, according to the PS categorization for subject areas, with some minor modifications, 14 different subject areas were developed. Therefore, the subject areas in alphabetical order were “behavior, breeding and quantitative genetics, education and extension, health and welfare, immunology, management and environment, metabolism and nutrition, microbiology and virology, modeling, molecular biology, physiology and anatomy, production, products, processing and marketing, and reproduction”. The term “modeling” may not independently be considered as a category. However, due to various keywords/phrases related to the “modeling” and importance of this term in the recent decade, “modeling” was included in content analyses as an independent category. Considering the consultation of expert poultry scientists in each subject area, the major items explored in the title, abstract, and keywords of all the 22,451 retrieved articles. The major items are alphabetically listed in Table 1. Some items were common in 2 or more subject areas. Moreover, some overlapped subject areas have emerged. For example, according to the frequency of overlapped items, the merged items were “health and welfare”, and “nutrition and metabolism”. Furthermore, some items were found from article title/abstract/keywords, while they were not capable to characterize based on PS subject area categorization. Therefore, some further subject areas were included such as “education and extension”, “modeling”, and “microbiology and virology” which were included in the list (Table 1).

**Table 1:** Poultry science subject areas.

Main subject areas	Major items
Behavior	Adaptability, Aggressiveness, Beak trimming, Behavior, Brain, Broodiness, Cannibalism, Cognition, Cognitive, Domestication, Fear, Feather pecking, Molting
Breeding and Quantitative Genetics	Biodiversity, best linear unbiased prediction (BLUP), Breeding value, Breeding, Common environmental effect, Cross-breeding, (Co)variance components, (Co)variance function, Direct genetic effect, estimated breeding value (EBV), genomic BLUP (gBLUP), genomic EBV (GEBV), Genetic parameters, Genomic prediction, GWAS, Hereditary, Heritability, Imputation, Inbreeding, Inheritance, Line-breeding, Maternal effect, Mixed linear model, Pedigree, Permanent environmental effect, quantitative traits loci (QTL) mapping, Random regression, Repeatability, Selection, Selective sweep, Signature of selection
Education and Extension	Attitude, Consumer, Careers, Council, Course, Education, Extension, Ethics, Graduate, Insight, “*Poultry Science*”, Research and development, Secretary, Student, University, Poultry industry
Health and Welfare	Care, Drug, Disease, Disinfection, Health, Hygiene, Illness, Medicine, Medicinal plants, Remedies, Sanitation, Syndrome, Veterinary, Welfare, Well-being
Immunology	Adaptive immunity, Antibody, Antigen, Basophils, B-cells, bursa of Fabricius, CD4, CD8, Cell-mediated immunity, Eosinophils, Heterophils, Humoral immunity, IgA, IgE, IgG, IgM, IgY, Immune, Immunity, Immunobiology, Immunocompetence, Immunogenetics, Immunoglobulin, Immunoinformatics, Immunological, Immunobiology, Immunology, Immunome, Immunomics, Infection, Innate immunity, Leukocytes, Lymph nodes, Lymphocytes, Macrophage, major histocompatibility complex (MHC), Monocytes, Natural immunity, Phagocytosis, Pathogen, sheep red blood cells (SRBC), T-Cells, Thymus, Vaccination, Vaccines, White blood cells (WBC)
Management and environment	Cages, Climate, Cold-stress, Environment, Farm, Floor, Free-range, Heat-stress, House, Light(ing), Management, Rearing system(s), Stress, sustainability
Metabolism and nutrition	Absorption, Aflatoxin, Amino acids (mainly Methionine, Lysine, Tryptophan, Threonine, Arginine), Anabolism, Barley, Bioavailability, Catabolism, Cereals, Corn, Crude energy, Crude protein, Diet, Dietary, Digestibility, Digestion, Digestive tract, Energy balance, Essential oil, Fatty acids (Linoleic, Linolenic, poly-unsaturated fatty acids (PUFA), Poly unsaturated fatty acids), Feed Additives, Feeds, Feeding, Gastrointestinal tract, Grains, Intake, Maize, Meal, Medicinal plants, Metabolic, Metabolism, Metabolizable, Minerals (mainly Ca, Cl, Fe, K, Li, Mg, Mn, P, S, Zn), Net Energy, Nutrients, Nutrition, Particle size, Phytase, Phytate, Prebiotics, Probiotics, Ration, Rice, Soybean, Supplementation, Synbiotics, Toxins, Vitamins (Vitamin A, Vitamin B complex, Vitamin C, Vitamin D, Vitamin E, Vitamin K), Wheat
Microbiology and virology^1^	16S rRNA, Antibacterial, Antibiotics, Antimicrobial, Avibacterium, Bacterial, Bacteriology, Bronchitis, Campylobacter, Chlamydia, Cholera, Clostridium, *E. coli, Eimeria, Enterococcus, Erysipelothrix*, Fowlpox, *Gallibacterium*, Gut bacteria, *Lactobacillus, Listeria*, Marek's Disease, Microbiology, Microbiome, Microbiota, *Morganella, Mycoplasma*, Newcastle, Prebiotic, Probiotic, Pseudomonas, *Salmonella, Shigella, Staphylococcus*, Symbiotic, *Toxoplasma*, Viral, Virology, Virulence, Virus
Modeling	ANN, Artificial neural network, Decision tree, Egg production curve, Functions, Fuzzy logic, Gamma function, Genetic algorithm, Gompertz, Growth curve, Growth pattern, Image processing, Logistic, Machine learning, Mathematical, Mathematics, Meta-analysis, Meta-regression, Models, Modeling, Modelling, Pattern recognition, Random Forest, Richards, Spline, von Bertalanffy, Woods function
Molecular biology	AFLP, Allele, Blotting, Centromere, Chromosome, Copy number variation (CNV), DNA, Epigenetics, Epigenome, Epigenomics, Exome, Exon, Finger printing, Gene, Gene expression, Gene(tic) network, Genome, Genomic(s), Genotype, Genotyping, Genotyping, Haplotype, Intron, Karyotype, Linkage map, Loci, Locus, Microsatellites, Molecular markers, PCR, Phylogenetic, Polymorphism, Protein network, Real-time (PCR), RFLP, RNA, RNA-Seq, Sequencing, Signaling pathway, Single nucleotide, SNP, SSCP, Telomer, Transcripts, Transcriptome
Physiology and anatomy	Adrenal, Artery, Blood, Blood/serum/plasma + biochemistry/biochemicals parameters, Bone, Cardia, Cell, Cloaca, Crop, -cyte, Derm, Digestive system, Endocrinology, Enzyme, Erythrocyte, Esophagus, Gizzard, Heart, Hematological parameters, Hepatocyte, Hormones, Intestinal, Kidney, Liver, Lung, Metabolites, Neonatal development, Neural system, Neuron, Osteo-, Osteocyte, Physiological, Physiology, Plasma, Serum, Spleen, Surgical, Thrombocyte, Vascular, Vein
Production	Body weight, Conventional production system, Egg laying, Egg, Eggshell, Growing, Growth, Laying hen, Organic production system, Production, Productive, Weight gain
Products, processing and marketing	Antioxidants, Breast, Carcass, Chicken fillet, Chicken Nugget, Chilling, Consumer, Cooking, Dressing, Market, Marketing, Meat, Processing, Shopping, Slaughter, Tenderness, Thawing, Yield
Reproduction	Caponization, Embryo, Estrogen, Fertile, Fertility, Fetal, Fetus, Gonad, Hatchability, Insemination, Mating, Maturity, Ovarian, Ovary, Oviduct, Oviposition, Ovulation, Ovum, Phallus, Reproduction, Reproductive, Semen, Sex Hormones, Sexing, Sexual, Sperm, Testicle, Testes, Testosterone, Vas Deferens

1 Given that different species of bacteria and viruses have been studied in PS, only the genus of them is mentioned in the Table 1.

#### Poultry Species/Strains

To determine each poultry occurrence in the publications in the last 100 yr in PS, the major species/breeds/strains were considered. During the past 100 yr different scientific names of each genus have been possibly studied; however, breeds/stains of each species were integrated and considered as their common name.

### Scientometrics

Using 22,451 articles published over 100 yr (1921–2020) in PS, primary data preparation was conducted through Bibexcel software (Bibexcel, 2013). Type of publications, number of authors and publications with/without citations were extracted. Moreover, using NetDraw (version 2.153), and UCInet (6.581 release) softwares (Borgatti et al., 2002), scientific collaborations (countries and authors) were retrieved. A minimum of 25 co-authors were involved for drawing author's co-operation networks. Moreover, the VOSviewer software was employed to draw the keywords co-occurrence density maps (Van Eck and Waltman, 2010). Density drawings were prepared to visualize “hot” spots and trends of the keywords in the published articles. Because there was variety between keywords with the same concept, the keywords were preprocessed and assessed, and we made them unique prior to the visualization. Thus, recommendations of poultry scientists have been utilized to the keyword's homogenization, classification, and analyses. For example, regardless of the virus name, different kinds of viruses with frequency less than 5 times were considered as “viruses”. The same manner was used for other phrases such as antibiotics, fatty acids, amino acids, genes, vitamins, and immunoglobins. For bacteria, only their genus was considered in density maps. For “trace elements” the expression “minor elements” was included, and for major elements their abbreviations (such as Ca and P) were included in density maps. Closer phrases in the density map reflect the more frequent co-occurrence keywords. Moreover, as frequency of keywords increased, the phrase letter became bigger and the background color in the density map tended to become red against less frequent keywords which would be seen in blue context and more little letters. Yellowish and green colors referred to the intermediate frequencies.

## RESULTS AND DISCUSSION

### Content Analyses

#### Number of Publications

**Figure 1:**
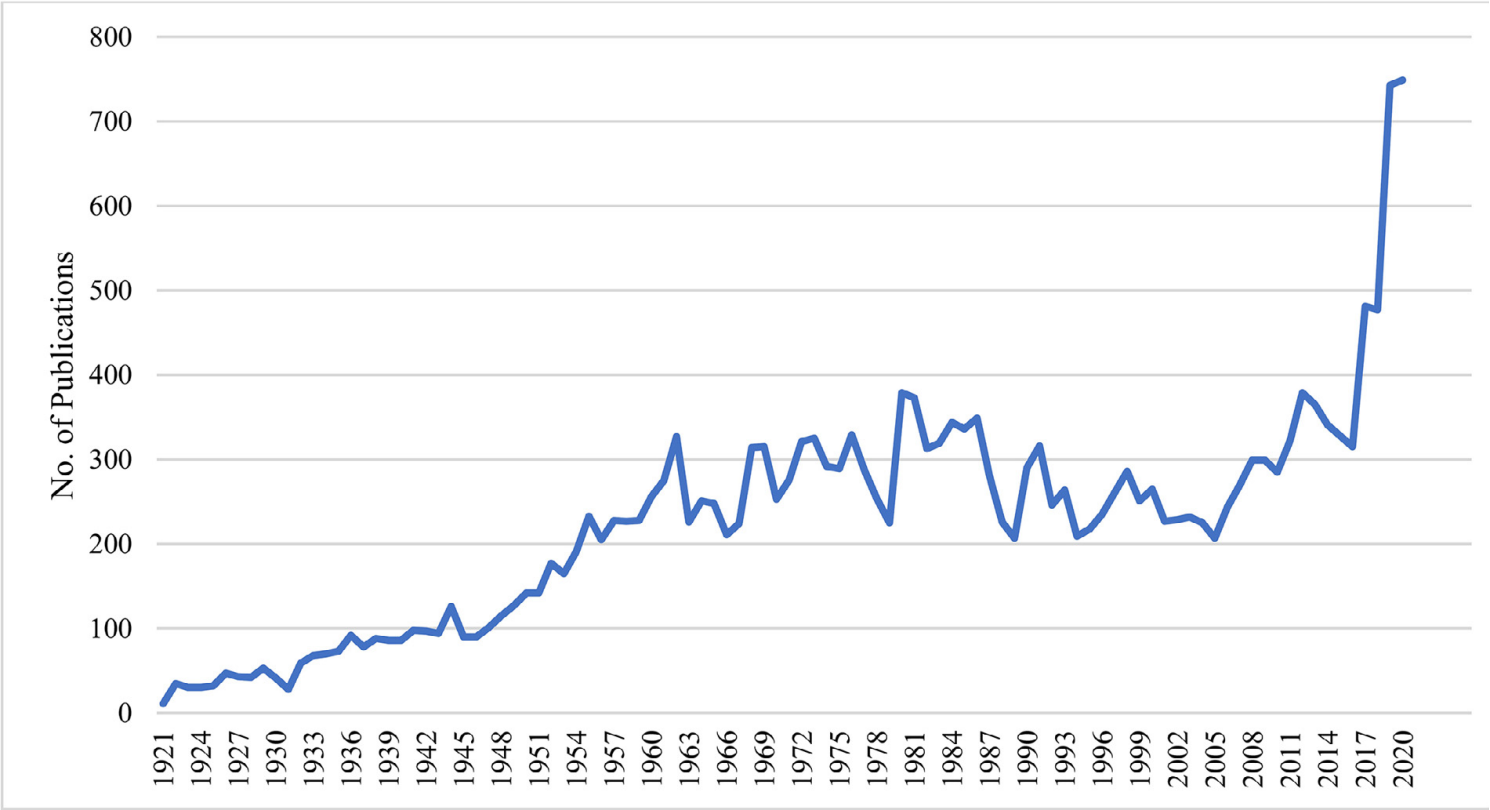
Publications in Poultry Science over past 100 yr.

Number of articles/years in the PS over 100 yR (1921–2020) are shown in Figure 1. According to the graph pattern, number of publications/years can be partitioned into 3 periods. In the first period from 1921 to 1960, the number of articles increased, while in the second period from 1961 to 2010 the number of articles/years rose and fluctuated. In the last period from 2011 to 2020, article publishing in PS accelerated rapidly whereas the number of published articles in the 2011–2020 time-period were more than 2 times compared with the previous periods. The number of articles in the last 5 yR (2016–2020) increased dramatically with 315, 481, 477, 743, and 749 articles/year, respectively.

#### Subject Areas

**Figure 2:**
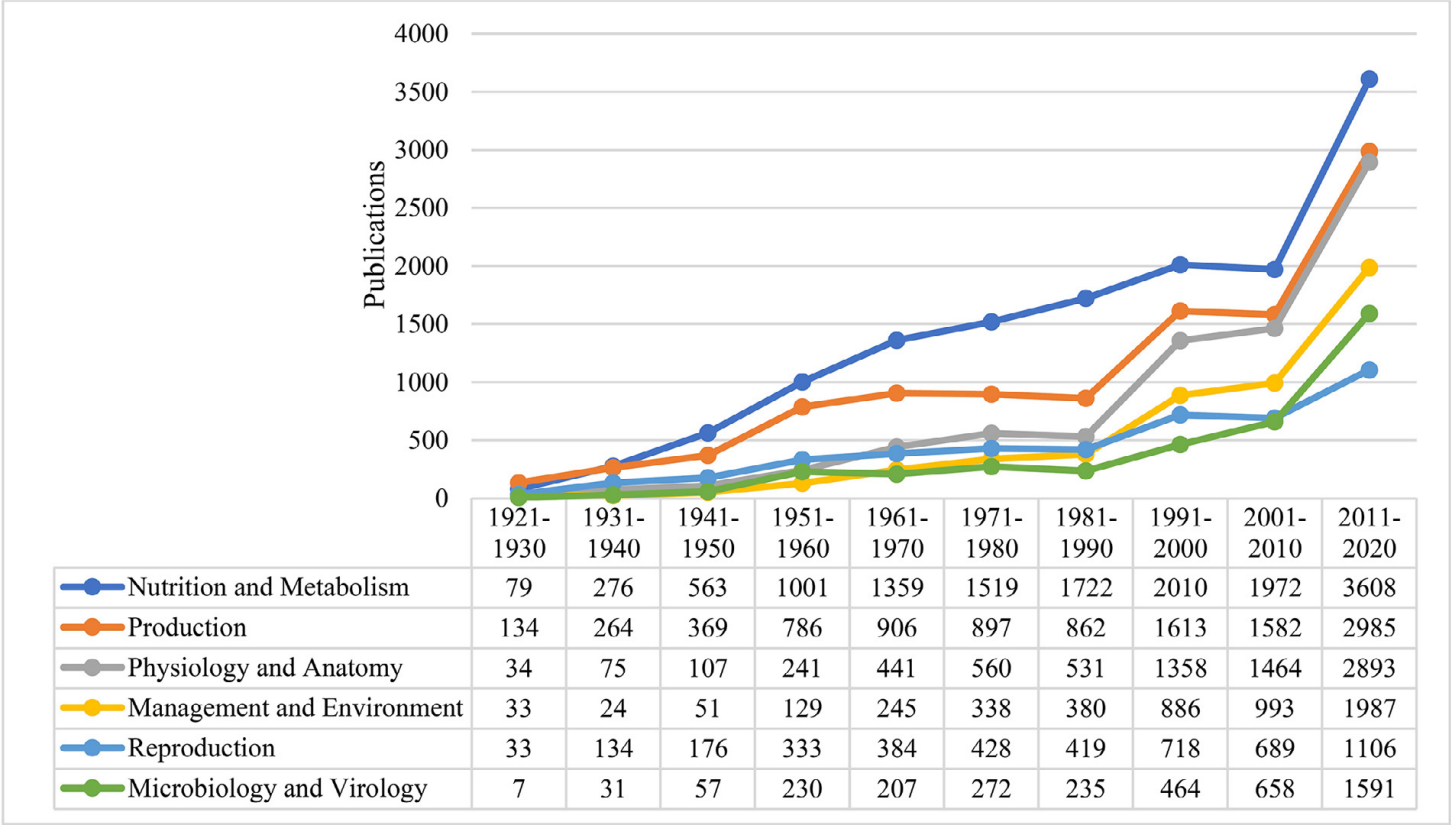
The most frequent subject areas published during the last 100 yr in Poultry Science.

Considering the 22,451 articles during 100 yr of publishing PS, the most frequent subject areas were “nutrition and metabolism” (14,109 articles), “production” (10,398), “physiology and anatomy” (7,704), “management and environment” (5,066), “reproduction” (4,420), and “microbiology and virology” (3,752; Figure 2). Other subject areas, which were not included in the Figure 2, were “products, processing, and marketing” (3,715 articles), “health and welfare” (3,702), “immunology” (3,512), “breeding and genetics” (3,505), “molecular biology” (2,466), “behavior” (1,664), “education and extension” (1,638), and “modeling” (1,114). With our disciplines in the current study, some papers may be categorized into more than one subject area. Therefore, sum of the articles in the 14 subject areas are more than total retrieved articles (22,451 articles). Frequency of subject areas refers to the fact that during the last 100 yr, poultry nutrition and metabolism were the most important research area covered in PS (Powers and Angel, 2008). In contrast, according to the published articles, modeling received less attention in the PS during 100 yr. Considering the 6 most frequent subject areas (Figure 2), all the areas showed the same pattern during different decades, except for reproduction. Among subject areas, “metabolism and nutrition” during 1990s, and “microbiology and virology” during 2000s received more attention than reproduction, reflecting the poultry nutrition (Lopez and Leeson, 1994; Powers and Angel, 2008) and health/immunity (Siegel and Honaker, 2009) importance in the recent few decades.

Considering the percentage of each subject area during 10 decades of publishing PS (from 1921–1930 decade to the 2011–2020 decade), different results are determined, especially in the last decade (2011–2020). According to the percentage of each subject area, the most important subject area in 2011–2020 period was molecular biology with 57.58%. In other words, more than half of the articles in the molecular biology subject area were published in the last 10 yr and the remaining 42.40% were published during 90 previous years (1921–2010). Besides, if subject areas over 100 yr were considered, the rank of molecular biology was 10 with 2,466 articles, while considering the last decade, surprisingly it ranks first between 14 subject areas. Followed by molecular biology, other subject areas based on their percentage in the 2011–2020 time-period were “modeling” (48.88%), “education and extension” (44.75%), “health and welfare” (44.59%), immunology (43.04%), “microbiology and virology” (42.40%), “management and environment” (39.23%), “physiology and anatomy” (37.56%), “products, processing and marketing” (36.85%), “breeding and genetics” (32.89%), “behavior” (29.63%), “production” (28.71%), and “nutrition and metabolism” (25.58%) [Sum of Percent deviates from 100 due to overlap of subject areas]. Surprisingly, while the “nutrition and metabolism” were calculated as the most frequent subject areas in 100 yr of PS publishing, it has been considered in only 25% of studies during 2011–2020, and almost 75% of articles in this area were published during 1921–2010 time-period. The other surprising result was assigned to the “modeling”, that ranks second in the last decade, while considering the entire 10 decades it become the less frequent (14th) subject area. In fact, almost half of the articles (48.88%) in the modeling subject area were published just in the 2011–2020 time-period. Entering the big-data era and utilizing the high-throughput technologies in biological sciences widely influenced the contribution of mathematical models to extract biological concepts from the raw data (Cogburn et al., 2007). Developing mathematical methods and employing artificial intelligence in the poultry science have become undeniable (Jahan et al., 2020). It seems that modeling remains one of the most attractive research areas for scientists at least in the upcoming future decades.

Genetics and breeding have overlapped with most of the subject areas. For instance, due to the feeding programs and dietary treatments, analysis of gene expression has become usual in the nutrition studies, which may entitle nutrigenetics/nutrigenomics. Moreover, poultry breeders have used feed efficiency as an economically important trait for estimation of genetic parameters (Varkoohi et al., 2011; Wolc et al., 2013). In the last recent decade, however, developing molecular genetics and molecular laboratory automated equipment provided more interesting situation for poultry geneticists (Guo et al., 2020). Overlapping genetics and immunology is also a considerable topic in poultry science (Kogut, 2009; Mohammadi-Tighsiah et al., 2018).

#### Poultry Species/Strains

**Figure 3:**
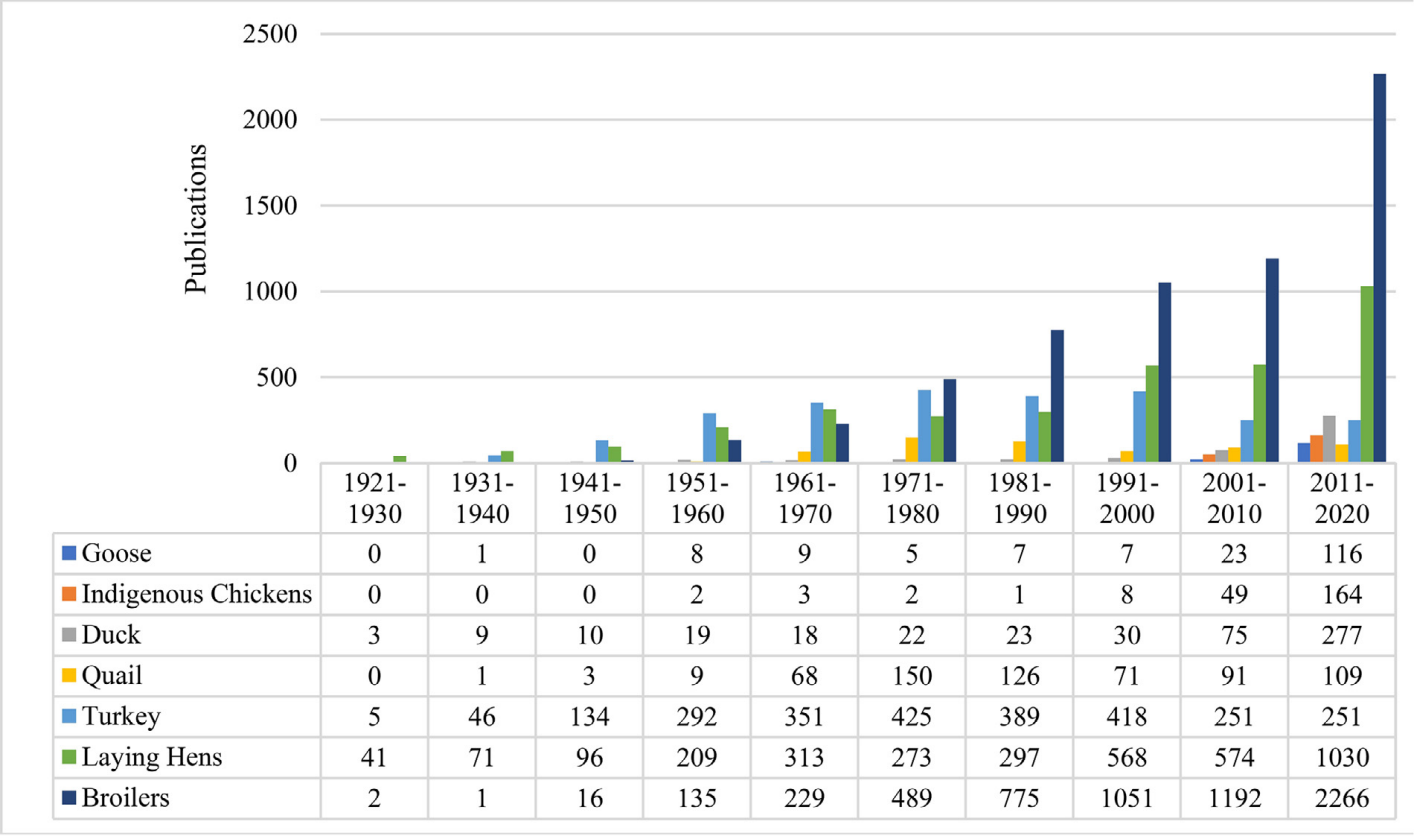
The most frequent poultry species considered during the last 100 yr in Poultry Science.

**Figure 4:**
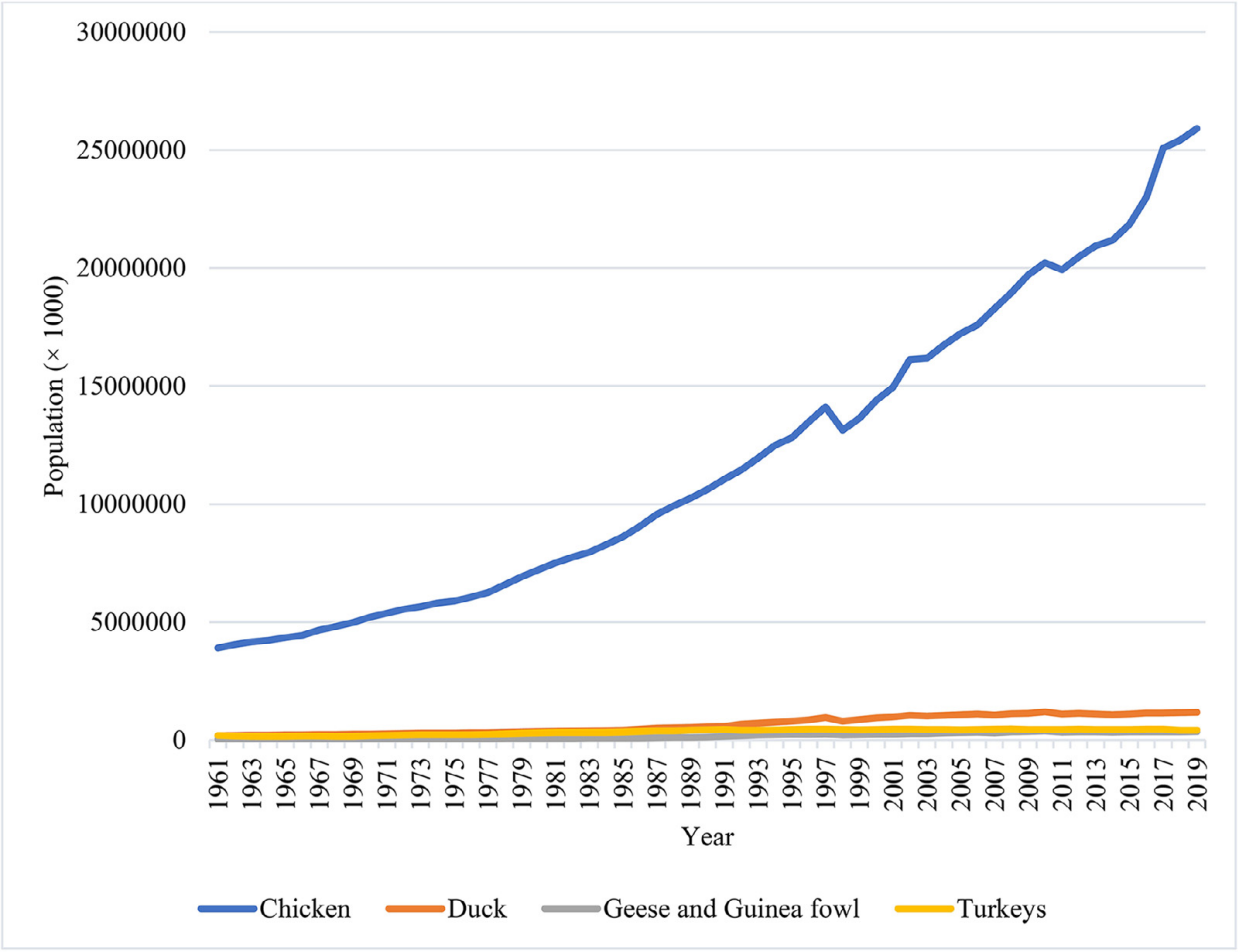
Population of the most popular poultry species in the world (× 1,000) (FAOSTAT Food and Agriculture Organization of the United Nations FAO, 2022).

Poultry species/breed/strain explored in retrieved articles were domestic chicken (*Gallus gallus domesticus*: broilers, laying hens, indigenous chickens), duck (*Anas platyrhynchos domesticus*), emu (*Dromaius novaehollandiae*), goose (*Anser anser*), guinea fowl (*Numida meleagris domestica*), ostrich (*Struthio camelus*), partridge (*Perdix perdix*), pheasant (*Phasianus colchicus*), pigeon (*Columba livia domestica*), quail (*Coturnix coturnix*), and turkeys (*Meleagris gallopavo*). The most frequent poultry species/strains retrieved from 22,451 published articles in PS were broilers (retrieved in 6,156 articles), laying hens (3,472), turkeys (2,562), quail (628), duck (486), indigenous chickens (229), goose (176), pigeon (82), pheasant (61), partridge (58), guinea fowl (34), ostrich (30), and emu (9), respectively (Figure 3). Until 1970s, number of articles on turkey and laying hens were more than broilers. However, during the last 50 yr, broilers have received more attention than other poultry species/strains, whereas in recent decade (2011–2020) the number of articles on broilers (2,266 articles) were more than twice the number of articles on laying hens (1,030), and more than 9 times higher than the number of articles on turkeys (251). Number of articles on turkeys over the last 2 decades were constant (251); however, they were lower than articles published during 1951–2000. Over 1970s, quail attracted more attention than other time-periods (150 publications). However, the number of publications on quails has decreased and revealed fluctuating trends during the last 40 yr. Domestic chicken (broilers, laying hens, and indigenous chickens) assigned for 9,857 articles (43.89% of total). Trends in increasing of live poultry population in the world (FAOSTAT Food and Agriculture Organization of the United Nations FAO, 2022) are shown in Figure 4. Results showed that population of ducks, geese and guinea fowls, and turkeys have increased during the last 60 yr, while increasing trend of chicken population was remarkable. Therefore, growing number of publications on domestic chicken may be a response to the globally growing population of this species and demand of poultry breeders to know more about them.

Indigenous chickens have received more attention during the last decade resulting set as more than 3 times compared with the 2001–2010 time-period (164 vs. 49 articles). Globally increased attention to the indigenous chickens may be associated, among others, with the importance of biodiversity in poultry populations (Fathi et al., 2017), increased susceptibility to the stressors (Mignon-Grasteau et al., 2015; Napper et al., 2015; Lauridsen, 2019), assessment to the indigenous chicken productive records involved to the genetic and phenotypic analyses (Ghorbani et al., 2013; Faraji Arough et al., 2019), and needs for introducing relevant strains for outdoor production systems (Stadig et al., 2016). Moreover, considering the last 2 decades, articles on duck and goose increased 3 to 5 times.

### Scientometrics

#### Countries’ Collaboration

**Figure 5:**
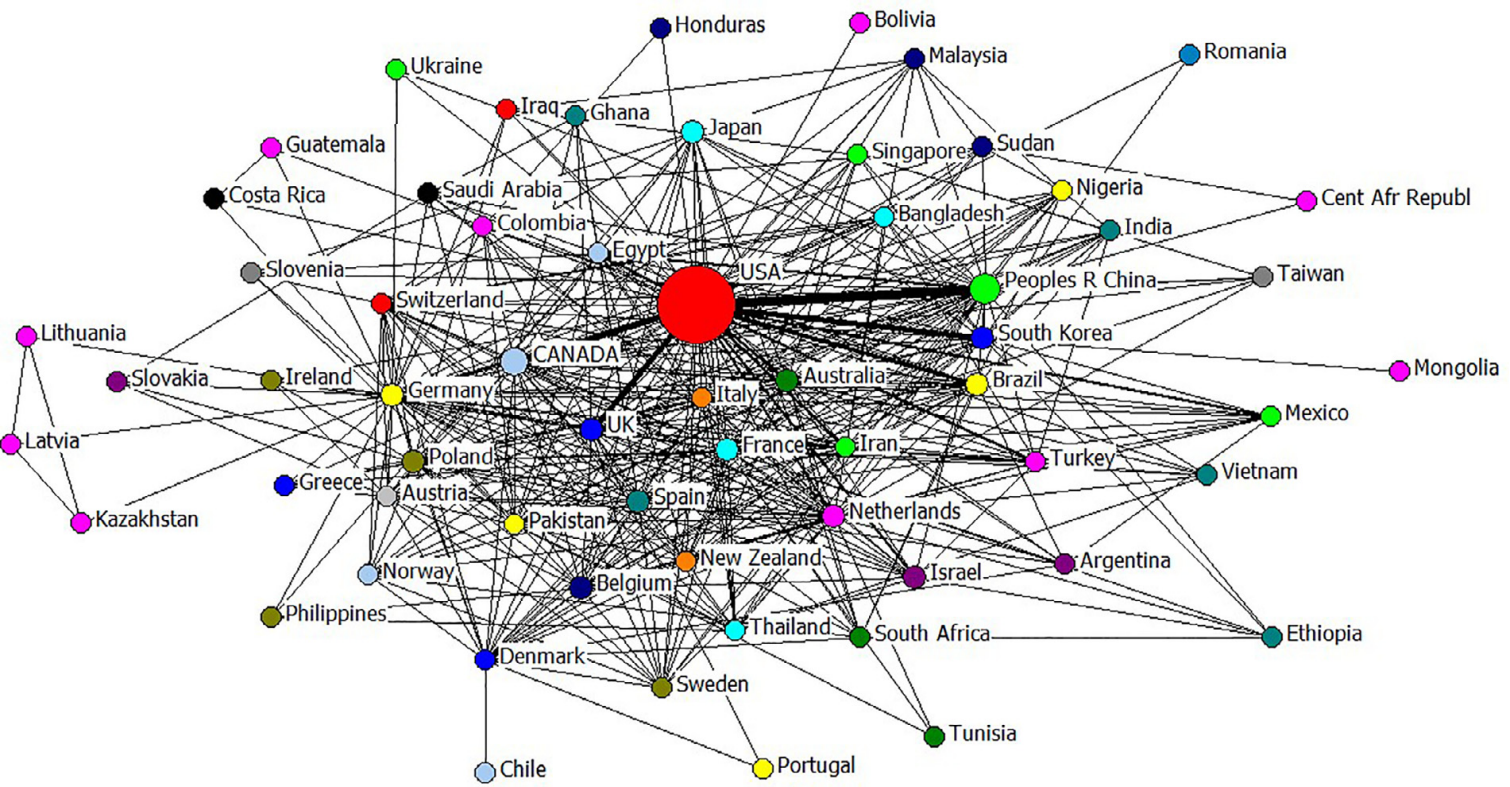
Scientific collaborations among countries that contributed to the publication of articles in Poultry Science during 100 yr (1921–2020).

**Figure 6:**
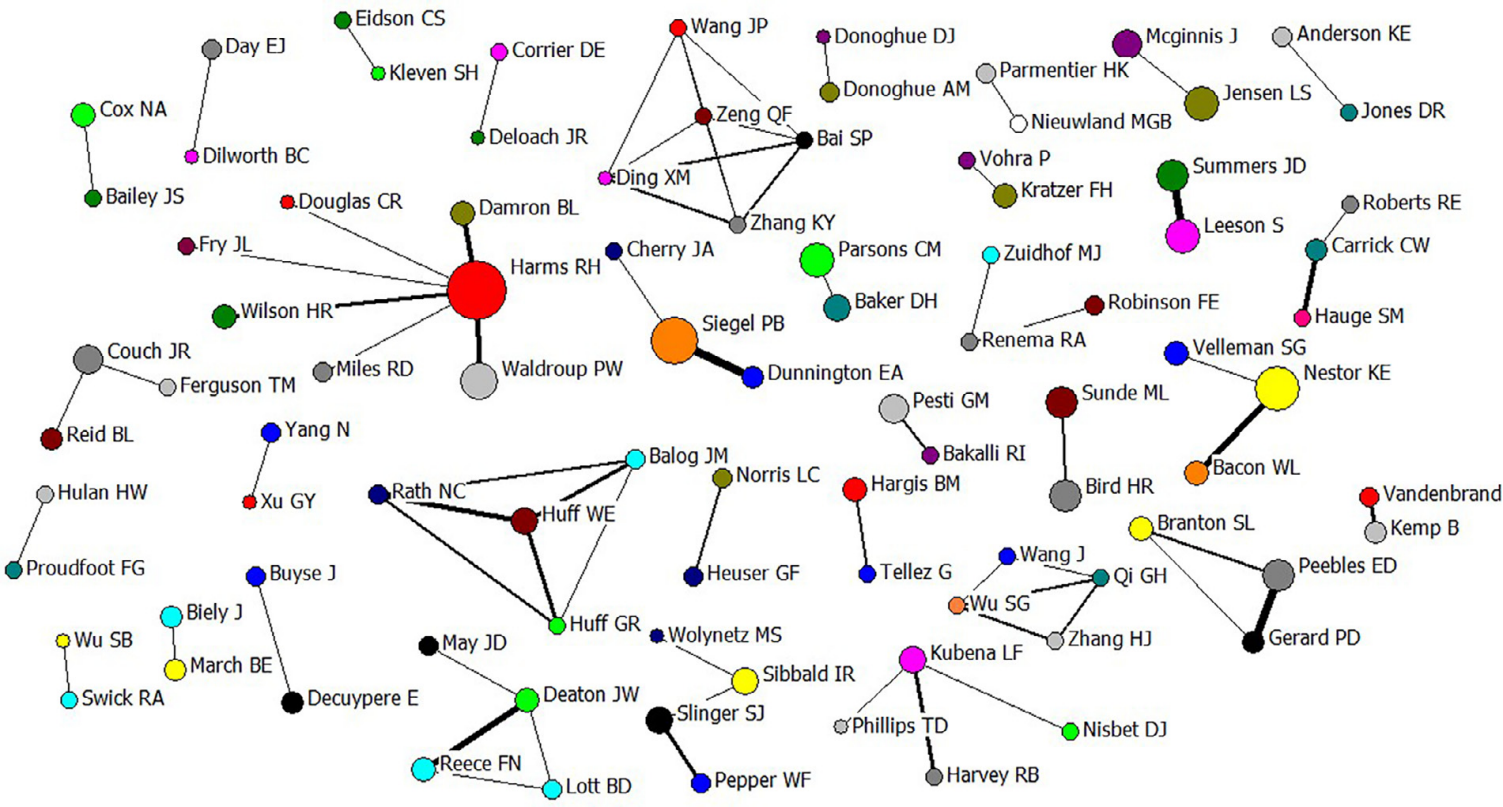
Scientific collaborations among authors (with at least 25 common articles) that contributed to publish in Poultry Science during 100 yr (1921–2020).

Totally, 108 countries contributed to publish in PS, however, all the articles were published in English language. To display among countries collaboration network, each node (circle) is representative of a country and lines between the nodes refer to the contribution among countries. Higher publications associated with a country resulted in wider diameter of the corresponding circle. Moreover, more collaboration between countries resulted in stronger connecting lines between the nodes (Figure 5). The same manner was used to develop among authors’ collaboration network (Figure 6). Using WoS database and information on the 21,826 articles (ignoring manually included articles), the most prolific country to publish articles in PS was United States (with 9,421 articles; 43.16% of total articles), followed by China (1,489; 6.82%), Canada (1,451; 6.65%), the Netherlands (450; 2.06%), Japan (378; 1.73%), Israel (342; 1.57%), France (331; 1.52%), Germany (320; 1.47%), Brazil (312; 1.43%), and South Korea (311; 1.43%), which were the top 10 countries, respectively. While United States, China, and Canada are the most prolific countries, connection among these countries are stronger than other countries.

The number of publications in the PS is not directly corresponding to the poultry population in each country. Based on its global widespread rearing (Figure 4), chicken (*Gallus gallus domesticus*) can be considered as index of the global poultry industry. Chicken population reared in the top 10 countries in addition with their rank and number of publications in the PS are shown in Table 2. United States with 9,222 m head of chicken stay on top and China (4,748 m) is the second country in the list. The rank of United States (1st), China (2nd), and somehow Brazil (4th) in chicken population is corresponding with their publications in PS. In other words, in these 3 countries the number of publications may reflect the need of poultry industry. Considering chicken population, Indonesia (3,560 m), Brazil (1,479 m), Pakistan (1,444 m), Iran (1,009 m), India (791 m), Mexico (591 m), Russia (497 m), and Viet Nam (410 m) were ranked third to 10th. While Canada was the third prolific country in PS, it ranks 29 with 172 m head chickens.

**Table 2:** Top ten countries at 2020 based on their chicken (*Gallus gallus*) population (FAOSTAT Food and Agriculture Organization of the United Nations FAO, 2022) compared with their rank in *Poultry Science* published articles during 1921–2020.

Country	Global rank of live chicken population	Chicken population (million head)	Number of publication in PS	Rank in PS
USA	1	9,222	9,421	1
China	2	4,748	1,489	2
Indonesia	3	3,560	19	51
Brazil	4	1479	312	9
Pakistan	5	1,444	98	25
Iran	6	1,009	168	17
India	7	791	88	28
Mexico	8	591	298	13
Russia	9	497	12	60
Vietnam	10	410	14	58

#### Authors’ Collaboration

The co-authors’ collaborations are shown in Figure 6. Based on WoS database, totally, 26,147 authors contributed to PS, with an average of 1.164 authors per publication. Among the authors, Harms RH (287 articles), Siegel PB (208), Nestor KE (199), Waldroup PW (156), and Parsons CM (150), published more than 150 articles in PS. Other most prolific authors with more than 100 papers in PS were Jensen LS, Leeson S, Brake J, Peebles ED, Sunde ML, Bird HR, Summers JD, Mcginnis J, Wideman RF, Pesti GM, Edwards HM, Couch JR, Stadelman WJ, Slinger SJ, and Kubena LF, with 146, 146, 141, 131, 126, 125, 125, 120, 120, 116, 115, 110, 107, 106, and 105 articles, respectively. Considering the collaboration between researchers, Siegel PB and Dunnington EA (71 articles), Gerard PD and Peebles ED (70), Leeson S and Summers JD (63), Deaton JW and Reece FN (60), Bacon WL and Nestor KE (56), Huff WE and Rath NC (53), and Damron BL and Harms RH (51), had more than 50 papers with each other in PS. It should be noted that a higher rate of publication did not ensure the author appearance in the network, because higher collaboration was the initial precondition (threshold = at least 25 collaborations; Figure 6). In addition to the authors’ collaboration to publish papers, citations to papers are important too. Recently the 100 most cited papers published in PS (1945 to 2020) were evaluated (Taylor, 2021). As reviewed by Taylor (2021), the most cited PS papers with more than 500 citations were published by Patterson and Burkholder (2003), Glick et al. (1956), Natt and Herrick (1952), Dibner and Richards (2005), and Sibbald (1976) with 742, 710, 683, 663, and 534 citations, respectively.

### Keyword Trends

**Figure 7:**
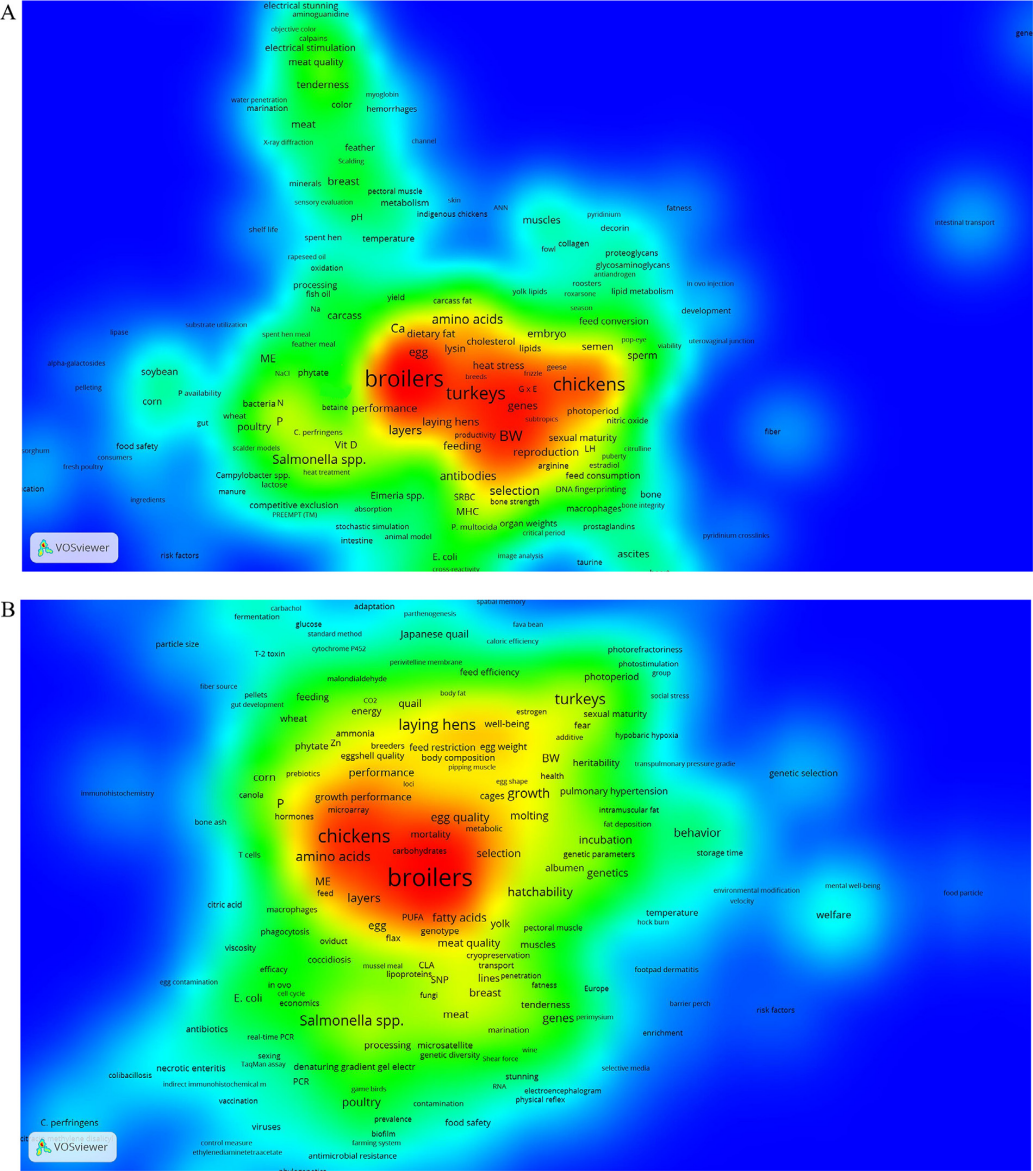

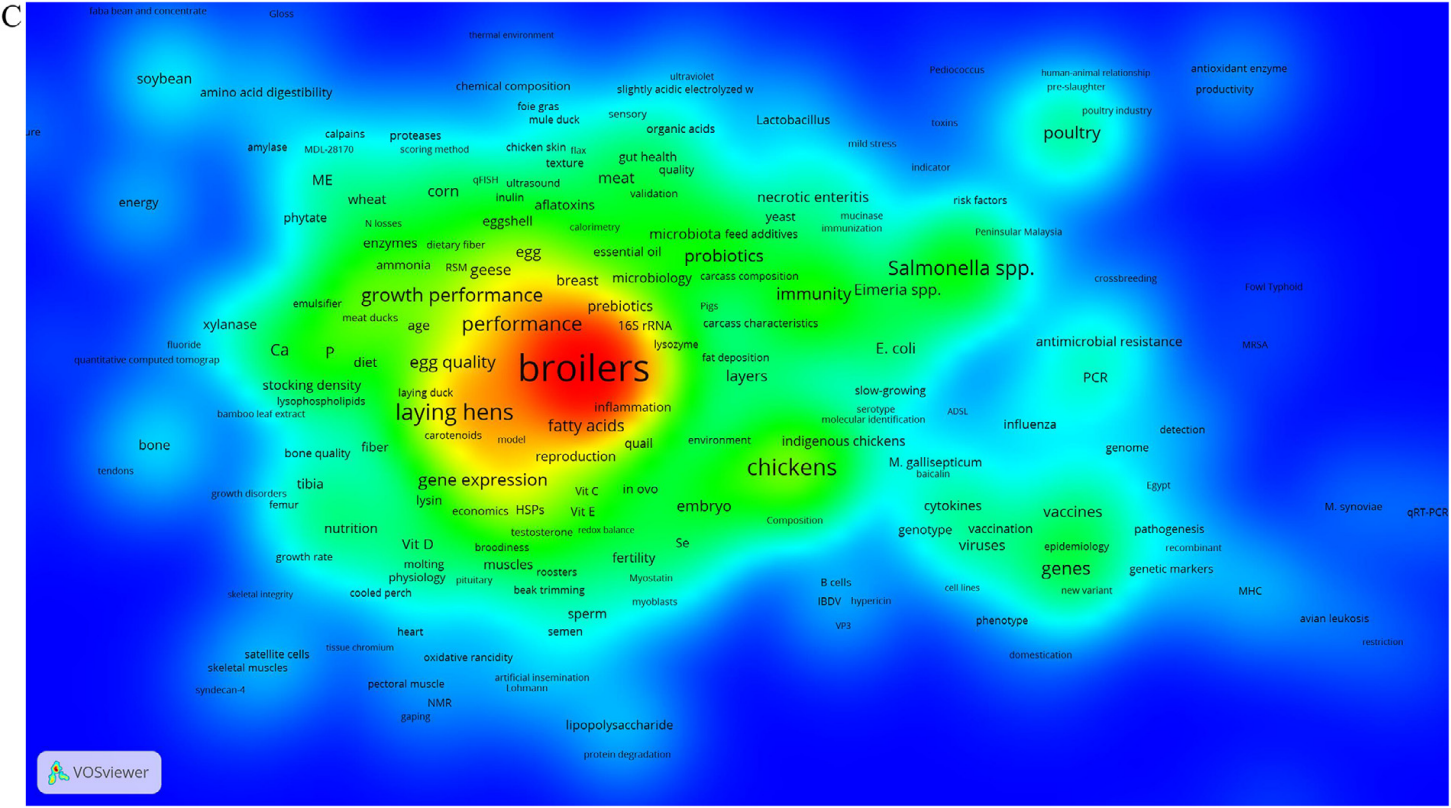
(A) = density map of keywords’ co-occurrence from 1921 to 2000; (B) = from 2001 to 2010; and (C) = from 2011 to 2020 (Threshold at least 10 co-occurred keywords).

To study the keyword co-occurred trends during 1921–2020, relative proportion of articles in each time-period were taken into account. Therefore, 3 time-periods have been considered: 1921 to 2000, 2001 to 2010, and 2011 to 2020 (Figures 7A–7C).

#### 1921–2000 Time-Period

The most frequent keyword in 1921–2000 time-period was broilers (373 times), followed by chickens (253), turkey (231), body weight (123), growth (123), and egg production (106) which repeated more than 100 times. Other frequent keywords were layers, hatchability, *Salmonella* spp., selection, poultry, broiler breeders, Ca, performance, P, and proteins. Before 2000, the most co-occurrence keywords were broilers and body weights (25 co-occurrence), body weight and egg production (18), ascites and broilers (16), Ca and P (16), broilers and growth (16), and turkey and growth (16). In other words, in the 1921–2000 time-period, “broilers” was the central keyword which occurred with a variety of other keywords such as ascites (16 times), growth (16), phytase (14), performance (14), body weight (13), tibial dyschondroplasia (12), *Salmonella* spp. (10), and P (10) (Figure 7A).

During the 1921–2000 time-period, computer simulation, artificial insemination, and genetic diversity were steadily getting more attention in published articles and have comparatively been considered as new comer topics in density map. In this time-period, beside broilers, turkeys, and chickens were the other important species/strains that included as co-occurred keywords in the articles, while the word “chickens” is widely referred to all of the new born birds’. Broadly, meat has become more important, while keywords such as meat, tenderness, breast, meat quality, pH, objective color, and myoglobin have started to be sharp in density map and appeared closely together, mostly in the green context. Really, in this time period developing slaughter technologies (Bilgili, 1999; Fletcher, 1999), and poultry meat processing (Sams, 1999) were mainly interested in PS. Antibodies, and sheep red blood cells (**SRBC**) are nominates of immune system performance which assumed major importance close to the central keywords. Humoral immunity and response to the vaccines in the modern chickens were challenging and experiments to study the immune system performance came into the spotlight (Bacon and Dietert, 1991; Chen et al., 1994; Dietert and Golemboski, 1998). Unlike meat and immunity, microbes were scattered mostly near the broilers and layers in the map. Ascites was also autonomously important in this time-period. Nutrition-related keywords including feed consumption, metabolism, feeding, metabolizable energy (**ME**), lysine, gut, intestine, and dietary fat were mostly scattered in whole density map. Nevertheless, corn and soybean as the main ingredients of the diet of the modern high-performing commercialized chickens emerged together. Obviously, at the end of the last century, the collaboration was formed between nutrition with immunology (Praharaj et al., 1997), health and disease (Klasing, 1998), genetics (Luiting and Urff, 1991), and reproduction (Meyer et al., 1980). Therefore, nutrition could be involved as a multidisciplinary issue in the PS during late 1921–2000 time-period.

Genetics-related keywords in 1921–2000 time-period had less variety than nutrition-related keywords. However, genetic by environment interaction (G × E), genes, and DNA fingerprinting was situated between chickens and turkeys in the red context, reflecting the importance of genetics. In the late 20th century, planning genetic strategies in commercial chicken lines became a major issue (Siegel and Dunnington, 1997). According to the patterns of density map, animal model, artificial neural networks (**ANN**), computer simulation, and image analysis were also marginally becoming more important. In this time-period, computer simulation was employed to plan genetic selection strategies (Muir, 1997). Moreover, artificial neural networks were first hired as predictive models (Roush et al., 1997; Cravener and Roush, 1999). Like genetic phrases, heat stress was located between central keywords. During late 20th century, challenges facing the poultry industry in the 21st century were predicted as reproduction, genetic selection, economic impact, and marketing (Hammerstedt, 1999).

#### 2001–2010 Time-Period

The most frequent keywords in the 2001–2010 time-period were broilers, chickens, and laying hens with 1159, 360, and 261 times, respectively. Followed by central keywords, the frequency of the performance, meat quality, growth performance, *Salmonella* spp., poultry, turkey, egg quality, and welfare were detected more than 100 times. Such 1921–2000 time-period, broilers and chickens were central keywords during 2001–2010, while turkey was separated. Moreover, laying hens was pushed to the margin from the red context compared with the previous time-period. Broilers mainly co-occurred with amino acids (56 times), and *Salmonella* spp. (28 times). In fact, it can be expected that nutrition and immunology/microbiology will be the most challenging issues in broiler production. (Maghsoudi et al., 2020).

It seems that egg quality, egg, and yolk tend to be substituted with the laying hens. On the way to specialization, pulmonary hypertension was also replaced with ascites. Similar to the 1921–2000 time-period, nutrition-related keywords including corn, wheat, canola, feed, ME, feeding, PUFA, phytate, and feed restriction were wholly scattered in the density map (Figure 7B). These nutritional keywords which mostly appeared in green context emphasize their general co-occurrence with other subject areas. The most cited paper of PS (728 times) which refers to the poultry nutrition have been published in this time period (Patterson and Burkholder, 2003). The immune-related phrases have become more professional and T-cells, phagocytosis, and monocytes appeared in the green context. In addition, with these phrases, authors utilize a variety of immune-related keywords and immunogenomics first became common (Lillehoj et al., 2007). However, due to the applied threshold in the current study, all the keywords were not included in the map. Nutritional immunology has also entered a new era (Kidd, 2004; Korver, 2006). Welfare and genetic selection can be considered as emerging fields in this time-period. In previous time-periods, meat-related phrases primarily co-occurred in the green context including meat quality, fatness, tenderness, breast, and pectoral muscle. At this time, meat was still an important keyword, maybe due to the importance of broilers (Barbut, 2009). The reproductive phrases first emerged in different locations in the map. The most important reproductive phrases were oviduct, sexing, sexual maturity, hatchability, and cryopreservation, which assume major importance alongside with other research fields. Among the most important studied subject areas (Table 1), reproduction has received less attention (Figure 2) based on the number of PS publications. However, Figure 7B refers to the most important co-occurred reproductive keywords. In addition, with the chickens and turkeys, quails were the other avian species which emerged in the 2001–2010 time-period. However, other species were not appeared in this time-period. *Salmonella* spp. And *E. coli* were the most important pathogens. They co-occurred with economics and antibiotics, imply their economic impact on the poultry industry, and critical needs to control them. Moreover, *Salmonella* spp. co-occurred with meat, and *E. coli* with egg, suggesting the more important ways of the food contaminations.

Rather than the first time-period, genetic-related phrases which were focused near the central keywords, in the 2001–2010 time-period they were scattered in the density map. The main genetic-related keywords were selection and microarray, which were located in the red zone. Selection co-occurred with broilers, as well as microarray with both chickens and growth performance. In fact, poultry geneticists tried to produce the most prolific chickens and broilers using genetic and breeding plans/techniques (Cogburn et al., 2003). In this time-period, the balance among different traits has been considered through genetic selection (Siegel and Honaker, 2009). Other genetic-related keywords were RNA, SNP, genes, genetic parameters, and heritability. The genetic-related keywords’ scattering could be related to the overlap of genetics with other research fields. In this time period, the phrase genomic(s) was broadly employed by poultry scientists (Lamont, 2006), and quantitative traits loci (**QTL**) mapping spend its golden duration (Abasht et al., 2006). However, due to the threshold applied in the density map (at least 10 co-occurred keywords), these phrases were not illustrated in the map.

#### 2011–2020 Time-Period

The most frequent keyword in the 2011–2020 time-period were broilers. Noticeably, “turkey” completely disappeared from the map (Figure 7C), and chickens established an independent island, while indigenous chickens were becoming neighborhood of chickens in green zone. The keywords “poultry” co-occurred with “human” and “industry” in a separate island, reflects the importance of human-animal relationship in poultry industry. Likewise, emerging new comer concepts in this area such as sustainability would be considerable. Rather than quail which emerged in 2001–2010 time-period, pigeon and geese are receiving importance in green context, while duck has stabilized its position in the red zone. In the 2011–2020 time-period, very close to the broilers, meat quality, and welfare were observed. Developing high-performing commercial broilers brought challenges such as performance and meat quality with 70 and 67 times co-occurrence with broilers, respectively. Moreover, poultry well-being and welfare has become challenging issues in the poultry industry during the last decade.

Immunity emerged close to the Eimeria, *E. coli*, and *Salmonella* spp. Moreover, other immune-related keywords such as B-cells are rare and emerged in the green zone. Considering Figures 7A–7C, *Salmonella* spp. Was one of the exceptional keywords which was presented during 100 yr of publishing PS (Cox, 1988; Revolledo et al., 2006; Ricke et al., 2013). Vaccines, vaccination, and cytokines also have established separate hot topics, close to epidemiology and viruses.

Although nutrition emerged marginally, nutritional keywords including digestibility, FCR, P, Ca, feed efficiency, fatty acids, methionine, lysine, gastrointestinal tract, gut, particle size, meal, and feed additives are wholly scattered in the map, particularly in the green zone. Related to nutrition, new comer phrases including toxins, probiotics, and antioxidants have also emerged. Behavioral phrases were revealed more than previous time periods, including molting, beak trimming, and feather pecking. It is noteworthy that here “beak trimming” was considered a behavioral phrase even not directly related to behavior but to a management practice, because the reason to apply that procedure relates to the behavior of the birds. The most important genetic-related keyword was “gene expression” in yellowish red zone. Such as 2001–2010 time-period, these types of keywords are totally scattered in the map. According to the Figure 2, the first subject area in the 2011–2020 time-period was molecular biology (while search in title, keywords, and abstract of the publications), however, this subject is not shown in the Figure 7C. The threshold for including keywords in the density map was at least 10 co-occurred keywords. Therefore, due to the variety in phrases included in molecular biology and/or genetics, it seems that the map did not demonstrate the importance of these subject area. However, some keywords including PCR, genome, and genes emerged marginally. Accompanying of genes with vaccines and epidemiology is remarkable (Gong et al., 2018).

## CONCLUSIONS

*Poultry Science* journal (PS) as a leader in poultry industry has a key role to enhance knowledge for both scientists and industry. “Nutrition and metabolism” as a subject area and “broilers” as a main poultry strain received more attention during the 100 yr in PS. Moreover, *Salmonella* spp. was important all the time. However, developing molecular biology methods and employing high-throughput technologies have resulted in producing big data in recent years. Therefore, to analyze these data, mathematical modeling seems to remain as research fronts in PS, besides “nutrition and metabolism” and “molecular biology”.

## DISCLOSURES

The authors have no conflict of interest.

## References

[bib0001] Abasht B., Dekkers J.C., Lamont S.J. (2006). Review of quantitative trait loci identified in the chicken. Poult. Sci..

[bib0002] Bacon L.D., Dietert R.R. (1991). Genetic control of cell-mediated immunity in chickens. Poult. Sci..

[bib0003] Barbut S. (2009). Pale, soft, and exudative poultry meat−reviewing ways to manage at the processing plant. Poult. Sci..

[bib0004] Beach J.R. (1921). The differential diagnosis of diseases of the head of fowls. Poult. Sci..

[bib0005] Bilgili S.F. (1999). Recent advances in electrical stunning. Poult. Sci..

[bib58] Borgatti S.P., Everett M.G., Freeman L.C. (2002).

[bib0006] Chen C.H., Gobel T.W., Kubota T., Cooper M.D. (1994). T cell development in the chicken. Poult. Sci..

[bib0007] Cogburn L.A., Porter T.E., Duclos M.J., Simon J., Burgess S.C., Zhu J.J., Cheng H.H., Dodgson J.B., Burnside J. (2007). Functional genomics of the chicken—a model organism. Poult. Sci..

[bib0008] Cogburn L.A., Wang X., Carre W., Rejto L., Porter T.E., Aggrey S.E., Simon J. (2003). Systems-wide chicken DNA microarrays, gene expression profiling, and discovery of functional genes. Poult. Sci..

[bib0009] Cox N.A. (1988). *Salmonella* methodology update. Poult. Sci..

[bib0010] Cravener T.L., Roush W.B. (1999). Improving neural network prediction of amino acid levels in feed ingredients. Poult. Sci..

[bib59] Dibner J., Richards J. (2005). Antibiotic growth promoters in agriculture: history and mode of action. Poult. Sci..

[bib0011] Dietert R.R., Golemboski K.A. (1998). Avian macrophage metabolism. Poult. Sci..

[bib0013] Faraji Arough H., Rokouei M., Maghsoudi A., Mehri M. (2019). Evaluation of non- linear growth curves models for native slow-growing Khazak chickens. Poult. Sci. J..

[bib0014] Fathi M.M., Al-Homidan I., Motawei M.I., Abou-Emera O.K., El-Zarei M.F. (2017). Evaluation of genetic diversity of Saudi native chicken populations using microsatellite markers. Poult. Sci..

[bib0012] FAOSTAT Food and Agriculture Organization of the United Nations (FAO). (2022) FAOSTAT database. Accessed Sep. 2022. http://faostat.fao.org/site/291/default.aspx.

[bib0015] Fletcher D.L. (1999). Slaughter technology. Poult. Sci..

[bib0016] Ghorbani S., Tahmoorespur M., Maghsoudi A., Abdollahi-Arpanahi R. (2013). Estimates of (co)variance components for production and reproduction traits with different models in Fars native fowls. Livestock Sci..

[bib0017] Glick B., Chang T.S., Jaap R.G. (1956). The Bursa of fabricius and antibody production. Poult. Sci..

[bib0018] Gong Q., Ruan M.D., Niu M.F., Qin C.L., Hou Y., Guo J.Z. (2018). Immune efficacy of DNA vaccines based on oprL and oprF genes of Pseudomonas aeruginosa in chickens. Poult. Sci..

[bib0019] Guo Y., Chai L., Aggrey S.E., Oladeinde A., Johnson J., Zock G. (2020). A machine vision-based method for monitoring broiler chicken floor distribution. Sensors.

[bib0020] Hammerstedt R.H. (1999). Symposium summary and challenges for the future. Poult. Sci..

[bib0021] Jahan M., Maghsoudi A., Rokouei M., Faraji-Arough H. (2020). Prediction and optimization of slaughter weight in meat-type quails using artificial neural network modeling. Poult. Sci..

[bib0022] Kidd M.T. (2004). Nutritional modulation of immune function in broilers. Poult. Sci..

[bib0023] Klasing K.C. (1998). Nutritional modulation of resistance to infectious diseases. Poult. Sci..

[bib0024] Kogut M.H. (2009). Impact of nutrition on the innate immune response to infection in poultry. J. Appl. Poult. Res..

[bib0025] Korver D.R. (2006). Overview of the immune dynamics of the digestive system. J. Appl. Poult. Res..

[bib0026] Lamont S.J. (2006). Perspectives in chicken genetics and genomics. Poult. Sci..

[bib0027] Lauridsen C. (2019). From oxidative stress to inflammation: redox balance and immune system. Poult. Sci..

[bib0028] Lillehoj H.S., Kim C.H., Keeler C.L., Zhang S. (2007). Immunogenomic approaches to study host immunity to enteric pathogens. Poult. Sci..

[bib0029] Lopez G., Leeson S. (1994). Nutrition and broiler breeder performance: a review with emphasis on response to diet protein. J. Appl. Poult. Res..

[bib0030] Luiting P., Urff E.M. (1991). Residual feed consumption in laying hens. 2. Genetic variation and correlations. Poult Sci..

[bib0031] Maghsoudi A., Vaziri E., Feizabadi M., Mehri M. (2020). Fifty years of sheep red blood cells to monitor humoral immunity in poultry: a scientometric evaluation. Poult Sci..

[bib0032] Martínez-López F.J., Merigó J.M., Valenzuela-Fernández L., Nicolás C. (2018). Fifty years of the European Journal of Marketing: a bibliometric analysis. Eur. J. Market..

[bib0033] Meyer G.B., Props C.F., Leighton A.T., Van Krey H.P., Potter L.M. (1980). Influence of dietary protein during the pre-breeder period on subsequent reproductive performance of large white turkeys. 1. Growth, feed consumption, and female-sex-limited reproductive traits. Poult. Sci..

[bib0034] Mignon-Grasteau S., Moreri U., Narcy A., Rousseau X., Rodenburg T.B., Tixier-Boichard M., Zerjal T. (2015). Robustness to chronic heat stress in laying hens: a meta-analysis. Poult. Sci..

[bib0035] Mohammadi-Tighsiah A., Maghsoudi A., Bagherzadeh-Kasmani F., Rokouei M., Faraji-Arough H. (2018). Bayesian analysis of genetic parameters for early growth traits and humoral immune responses in Japanese quail. Livestock Sci..

[bib0036] Muir W.M. (1997). Genetic selection strategies: computer modeling. Poult. Sci..

[bib0037] Napper S., Dadgar S., Arsenault R.J., Trost B., Scruten E., Kusalik A., Shand P. (2015). Induction of tissue- and stressor-specific kinomic responses in chickens exposed to hot and cold stresses. Poult. Sci..

[bib0038] Natt M.P., Herrick C.A. (1952). A new blood diluent for counting the erythrocytes and leucocytes of the chicken. Poult. Sci..

[bib0039] Patterson J.A., Burkholder K.M. (2003). Application of prebiotics and probiotics in poultry production. Poult. Sci..

[bib0040] Poultry Science Association. 2021. About PSA. Accessed Sep. 2022. https://poultryscience.org/.

[bib0041] Poultry Science. 2021. About the Journal. Accessed Sep. 2022. https://www.sciencedirect.com/journal/poultry-science/about/aims-and-scope.

[bib0042] Powers W., Angel R. (2008). A review of the capacity for nutritional strategies to address environmental challenges in poultry production. Poult. Sci..

[bib0043] Praharaj N.K., Dunnington E.A., Gross W.B., Siegel P.B. (1997). Dietary effects on immune response of fast-growing chicks to inoculation of sheep erythrocytes and *Escherichia coli*. Poult. Sci..

[bib0044] Revolledo L., Ferreira A.J.P., Mead G.C. (2006). Prospects in *Salmonella* control: competitive exclusion, probiotics, and enhancement of avian intestinal immunity. J. Appl. Poult. Res..

[bib0045] Ricke S.C., Khatiwara A., Kwon Y.M. (2013). Application of microarray analysis of foodborne *Salmonella* in poultry production: a review. Poult. Sci..

[bib0046] Roush W.B., Cravener T.L., Kirby Y.K., Wideman R.F. (1997). Probabilistic neural network prediction of ascites in broilers based on minimally invasive physiological factors. Poult. Sci..

[bib0047] Sams A.R. (1999). Meat quality during processing. Poult. Sci..

[bib0048] Scientific Journal Ranking. 2020. Poultry Science: Accessed Sep. 2022. https://www.scimagojr.com/journalsearch.php?q=36431&tip=sid&clean=0.

[bib0049] Sibbald I.R. (1976). A bioassay for true metabolizable energy in feedingstuffs. Poult Sci..

[bib0050] Siegel P.B., Dunnington E.A. (1997). Genetic selection strategies—population genetics. Poult. Sci..

[bib0051] Siegel P.B., Honaker C.F. (2009). Impact of genetic selection for growth and immunity on resource allocations. J. Appl. Poult. Res..

[bib0052] Singh S., Dhir S., Das V.M., Sharma A. (2020). Bibliometric overview of the Technological Forecasting and Social Change journal: analysis from 1970 to 2018. Technol. Forecast.Soc. Change.

[bib0053] Stadig L.M., Rodenburg T.B., Reubens B., Aerts J., Duquenne B., Tuyttens F.A. (2016). Effects of free-range access on production parameters and meat quality, composition and taste in slow-growing broiler chickens. Poult. Sci..

[bib0054] Taylor R.L. (2021). The 100 most cited Poultry Science papers. Poult. Sci.

[bib0055] van Eck N.J., Waltman L. (2010). Software survey: VOSviewer, a computer program for bibliometric mapping. Scientometrics.

[bib0056] Varkoohi S., Pakdel A., Moradi Shahr Babak M., Nejati Javaremi A., Kause A., Zaghari M. (2011). Genetic parameters for feed utilization traits in Japanese quail. Poult. Sci..

[bib0057] Wolc A., Arango J., Jankowski T., Settar P., Fulton J.E., O'Sullivan N.P., Fernando R., Garrick D.J., Dekkers J.C. (2013). Pedigree and genomic analyses of feed consumption and residual feed intake in laying hens. Poult. Sci..

